# A Prospective Study on Changes in Nutritional Status and Growth Following Two Years of Ketogenic Diet (KD) Therapy in Children with Refractory Epilepsy

**DOI:** 10.3390/nu11071596

**Published:** 2019-07-14

**Authors:** Marisa Armeno, Antonella Verini, Mariana del Pino, Maria Beatriz Araujo, Graciela Mestre, Gabriela Reyes, Roberto Horacio Caraballo

**Affiliations:** 1 Department of Clinical Nutrition, Hospital de Pediatría J.P. Garrahan, C1245AAM Buenos Aires, Argentina; 2 Department of Growth & Development, Hospital de Pediatría J.P. Garrahan, C1245AAM Buenos Aires, Argentina; 3 Food Services Area, Hospital de Pediatría J.P. Garrahan, C1245AAM Buenos Aires, Argentina; 4 Department of Neurology, Hospital de Pediatría J.P. Garrahan, C1245AAM Buenos Aires, Argentina

**Keywords:** ketogenic diet, refractory epilepsy, growth, nutritional status, children

## Abstract

Introduction: Epilepsy is a neurological disorder characterized by an increased susceptibility to seizures. The ketogenic diet (KD) is currently the most important alternative non-pharmacological treatment. Despite its long history of clinical use, it is not clear how this diet affects longitudinal growth in children. Methods: A prospective study was designed to evaluate growth and nutritional status in 45 children on KD. Growth was assessed by measuring weight, height, and body mass index (BMI). Standard deviation scores (SDS) were calculated for all measurement parameters at KD initiation and at a two-year follow-up. Results: Overall, 45 patients who completed 24 months on KD were enrolled. Median age was 6.6 years (0.8 to 17.3), with a male predominance (*n* = 23); 74% of the 45 patients were responders on seizure reduction at three months; 26% of patients were non-responders. In our study, using −1 SDS as a cut-off point, growth deceleration was observed in 9% (n: 4) of the patients; however, the nutritional status was maintained or even improved. No correlation with age, sex, or ambulatory status was found. Conclusions: The nutritional follow-up of these patients was helpful to improve overweight and thinness but could not avoid growth deceleration in some of them. These findings confirm that children with refractory epilepsy on KD treatment require careful growth monitoring.

## 1. Introduction

Epilepsy is a neurological disorder characterized by an increased susceptibility to seizures. The ketogenic diet (KD) is currently the most important alternative non-pharmacological treatment. Despite its long history of clinical use, it is not clear how this diet affects longitudinal growth in children [1]. This is particularly important as the KD is often used as a long-term therapy in different epileptic syndromes [2,3,4], such as Dravet syndrome, epilepsy with myoclonic-atonic seizures, Lennox–Gastaut syndrome.

In children, adequate growth and development are often used as markers for good health. Growth is controlled by a complex system of interactions between genetic and environmental factors. Any cause of malnutrition can result in growth attenuation in children [5], and conversely, linear growth of children (increase in height) is a valid indicator of nutritional adequacy over time.

The KD consists of food and/or formulas that are high in fat and very low in carbohydrates with controlled amounts of proteins, designed to induce a continuous state of ketosis [6]. Given that children may remain on the KD for a long time [7], the diet must provide adequate nutrition for optimum growth and development.

There is a need in clinical practice for continuous monitoring of the nutritional status of children during KD treatment, making continuous adjustments in caloric intake and supplementation with micronutrients and minerals to match the growth needs of the children because of the unbalanced nature of the KD. The ketogenic diet is typically discontinued after two or three years; however, in some children, the KD can be extended for more than 10 years. As more children are being treated for longer periods of time, the effects of the diet on health and growth must be addressed [8]. 

There are mixed data on the effect of the KD on growth in children [7,8,9,10,11,12,13]. Nevertheless, studies with a follow-up of more than 6 months suggest that the classic KD has negative effects on growth and may cause height deceleration over time [9].

To draw more solid conclusions on the growth of children on KD, a minimum follow-up period of two years is necessary [10]; however, almost all prospective studies published to date have shorter follow-ups. 

The aim of this study was to describe the growth pattern in height and weight of pediatric patients with refractory epilepsy on KD over a 24-month follow-up. The study is part of a larger prospective study that addresses the long-term effects of KD in children with epilepsy.

## 2. Materials and Methods 

The children with refractory epilepsy reported in this article are part of an ongoing prospective cohort to evaluate the long-term effects of the KD. Of 151 children recruited between 2012 and 2016, 45 stayed on KD for at least two years. The children were between 0.8 and 17.3 years of age (mean 6.6), and 23 were boys. They were followed-up to evaluate growth and nutritional status while on KD. The study was conducted at Hospital de Pediatría J.P. Garrahan in Buenos Aires, Argentina.

KD administration followed a modified version of the protocol of the Johns Hopkins Hospital [6]. The classical KD consisted of 90% of energy from fat and 10% of energy from protein plus carbohydrate and was supplemented with sugar-free multivitamin and calcium and potassium citrate supplements. The diet provided adequate protein intake (1 g/kg body weight per day) and adequate energy (RDA, Recommended Dietary Allowance) [14]. 

A safe protein-to-energy ratio was calculated by the dietitians according to Nation et al. [15]. Patients were encouraged to drink unrestricted amounts of sugar-free fluids. The majority of children were fed using solid food, and those unable to eat by mouth were fed the ketogenic formula through nasogastric or gastrostomy tubes.

Monitoring of the patients involved weekly clinical and laboratory follow-up during the first month and every three months thereafter, with control of ketosis, food recalls, and evaluation of compliance, as described in the consensus guidelines [16]. 

The caloric content and ratio of the diet were readjusted during the treatment for each child, to respond to problems of weight gain or loss and to optimize ketosis to control seizures.

Height and weight were measured during follow-up. All patient measurements were taken and followed-up by a trained observer during the entire study period with standardized anthropometric techniques. Height was measured with a stadiometer with 0.1 cm divisions, and weight with scales, with a scale accurate to 0.1 kg [17]. Body mass index (BMI) was calculated with the formula weight/height^2^. Individual growth curves of height and weight were plotted on national reference charts [18]. 

Height (zH), weight (zW), and BMI (zBMI) standard deviation scores (SDS) for age and sex were estimated using WHO [19] and local references [18], using the LMS growth program [20] at KD initiation and during the two-year follow-up. The SDS expresses the difference between the measurement of the individual and the mean value of the reference population as a proportion of the SDS of the reference population.

Individual growth curves were analyzed. In each child, growth retardation was defined as a deflection in height or weight of −1 SDS or more (delta between 12 months vs baseline and between 24 vs 12 months) as described by Grote et al. [21]. 

BMI was calculated for all children to assess the nutritional status. BMI was used to calculate the rates of thinness, overweight, and obesity, as defined by BMI SDS: thinness grade 3: −3 SDS, thinness grade 2: −2 SDS, thinness grade 1: −1SDS, normal weight: 0, overweight: +1 SDS, and overweight: +2 SDS [22,23].

Treatment efficacy was evaluated by calculating the percent reduction in seizure frequency from baseline (<50% reduction, 50–90% reduction, or >90% reduction) or seizure freedom based on the patient’s family self-report prior to KD initiation and at each follow-up visit.

The protocol was approved by the hospital institutional review board, and informed consent to the use the anthropometric data was obtained from a parent or caregiver.

For the statistical analysis, the STATISTIX8 software program was used. The student’s *t* test was used to compare z scores for weight, height, and BMI at baseline and at 24 months. The unpaired t test was used to compare mean z-score values for girls versus boys and ambulatory versus non-ambulatory groups. Ambulatory status was defined as being able to walk aided or unaided. The Wilcoxon signed-rank test was used for variables with a non-normal distribution. Categorical variables were analyzed using the chi-squared and Fisher’s exact tests. A two-tailed test was performed, and a *p* < 0.05 was considered significant.

## 3. Results

The clinical characteristics of the 45 children with refractory epilepsy on KD (23 males, 22 females), mean age 6 years and 6 months (range, 0.8–17.3 years), are presented in Table 1.

All patients were put on the classical KD with a 4:1 ratio, except for some patients, who received lower ketogenic ratios (two patients 3:1, two patients 2:1). The majority of children were fed with solid food; only seven received the ketogenic formula through a nasogastric or gastrostomy tube.

Regarding KD-related adverse effects, 3 patients developed acidosis, 5 constipation and 22 dyslipidemia. Thirteen children lost weight during the induction phase of the KD. Sixteen children had low vitamin D levels, and one had a zinc deficiency, two a copper deficiency, and nine a selenium deficiency. One patient had low magnesium levels, and three had hypocalcemia. All adverse effects were reverted with supplements and caloric adjustments according to the guidelines. Betahydroxybutyrate (β-OHB) levels were measured in 31/45 patients. The mean values were 3.2 mmol/L. In only three of our patients, mean β-OHB was <2 mmol/L during treatment. None of the patients had β-OHB levels above 5 mmol/L.

At nutritional evaluations during the follow-up, improvement of mean BMI SDS was observed, changing from −0.26 ± 1.5 (range, 5.2 to 2.4) to 0.01 ± 1.2 (range, −2 to 2.6).

**Figure 1:**
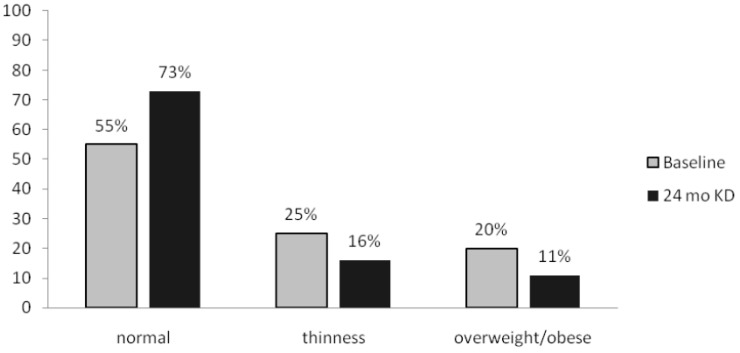
Nutritional status according to BMI at baseline and after 2 years on KD (%).

At KD initiation, BMI was normal in 55% of the patients, thinness was observed in 25%, and overweight or obesity in 20%. After 24 months of follow-up, 73% of the patients had a normal BMI, 16% were thin, and 11% were overweight/obese (see Figure 1).

**Figure 2:**
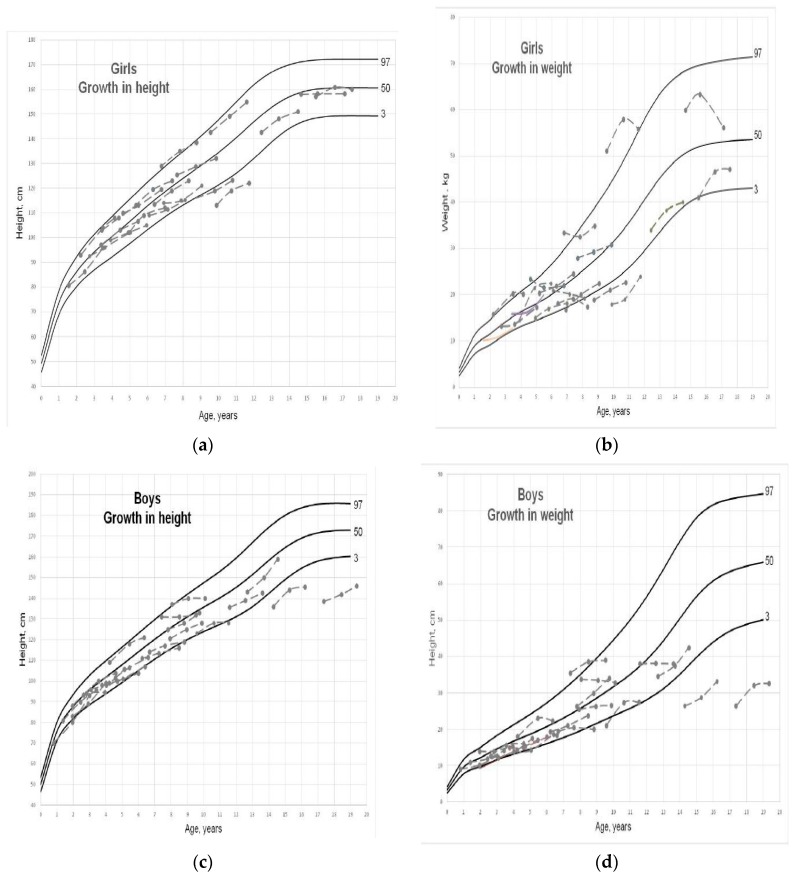
Individual growth curves showing height-for-age (median SDS) in girl (**a**). weight-for-age (median SDS) in girls (**b**), height-for-age (median SDS) in boys (**c**), weight-for-age (median SDS) in boys (**d**).

The height and weight growth curves for boys and girls are shown in Figure 2. When considering weight, its growth was below percentile 3 for two boys and two girls and above percentile 97 for one boy and one girl, while for all the other children it was within the normal range. Median SDS weight (max/min) at baseline was −0.6 (2.4/−5.2) and −0.11 (2.7/−3.5) for boys and girls, respectively. Normal growth was observed in most of the children during follow-up with a median SDS weight (max/min) at 2 years on KD of −0.86 (1.5/−3.5) and −0.4 (1.9/−3.0) for boys and girls, respectively.

Regarding height, its growth was below percentile 3 for two boys and two girls, while it was within the normal range for all the other children. Median SDS height (max/min) at baseline was −0.69 (2.0/−4.7) and −0.23 (2.1/−3.0) for boys and girls, respectively. Normal growth was observed in most of the children during follow-up, with a median SDS height (max/min) at 2 years on KD of −0.67 (0.97/−3.2) and −0.42 (1.7/−2.9) for boys and girls, respectively.

Table 2 shows the median changes in height and weight SDS over time. The mean delta SDS for weight and height were similar (approximately 0.26 and 0.34 SDS, respectively) during the first and the second year of follow-up. 

For weight, the analysis of individual growth records showed that only three boys and three girls had growth retardation, defined by a deflection of −1 SDS or more, during the first year on KD, and only one boy and two girls presented growth retardation during the second year of follow-up.

For height, the analysis of individual growth records showed that only two boys and one girl had growth retardation during the first year on KD, and only one boy had growth retardation during the second year of follow-up.

The types of epilepsy in this series of patients were Lennox–Gastaut syndrome in 10 children, epilepsy with myoclonic-atonic seizures (EMAS) in eight, symptomatic epilepsy in seven, West syndrome in six, febrile infection-related epilepsy syndrome (FIRES) in three, perinatal epileptic encephalopathy in two, continuous spike and wave during slow sleep (CSWS) in three, and encephalitis-related epilepsy in one.

Regarding the effectiveness of the KD, all patients received the classical KD, and 4% (2/45) of the patients became seizure-free, 26 % had a >90% seizure reduction, 43% had a 50–90% seizure reduction, and 27 % had a <50% seizure reduction.

## 4. Discussion

In this study, we evaluated growth in a subgroup of a cohort of patients that are being prospectively followed-up to determine the long-term effects of KD treatment for refractory epilepsy on growth and nutritional status.

In our series of 45 patients, after 24 months on KD, linear growth decreased more than −1 SDS in four patients (only in one patient at the two-year follow-up). No correlation with normal baseline height, age, or ambulatory status was found. Therefore, the majority of the children grew well, and the nutritional status, evaluated by mean BMI, at 24 months improved.

Nevertheless, BMI may not be a good parameter to measure the effect of KD in children. A simultaneous drop in a child’s weight and height would probably not manifest any changes in the BMI Z-score, while increased weight together with a halted or decreasing height may increase the index. Indeed, in our series, BMI improved after 24 months on KD, while good nutritional status was maintained in all patients. Groleau et al. [9] found similar results at 15 months of follow-up.

Previous studies assessing weight and height [8,11,12,13,24] used protocols with a calorie restriction of 75% of the RDA. In our study, no calorie restriction was used and even so, in three patients, weight was significantly affected by the diet after 24 months.

There are conflicting reports on how the KD affects linear growth of children on the diet [10]. Williams et al. [11] retrospectively evaluated 21 children treated with KD and reported height percentiles to fall from baseline in 18 children (86%) after 24 months on the diet. Peterson et al. [24] also found height Z-score to show a significant decrease between 6 to 12 months on KD. Additionally, Neal et al. [10] found a more marked drop in height in boys.

In agreement with our results, Kim et al. [25] found no changes or even improvements in mean BMI Z-scores. Vining et al. [8] prospectively assessed the growth of children treated with the KD over 24 months and found that the height Z-score declined after 6 months on KD. These authors found that only younger children (not older ones) fell more than 2 SDS below the mean height after three years on the diet. Nevertheless, in our study, no differences were found when considering age.

Spulber et al. [26] examined the effect of the KD on linear growth and IGF-1 levels in children with refractory epilepsy. They found that during the first year after diet initiation, growth velocity decreased significantly to a median of −4 SD, causing a drop in height SDS that correlated negatively with blood beta-hydroxybutyrate levels (BHB) levels and positively with serum IGF-1.

In our study, when individual anthropometric records were analyzed, only four children had a growth deceleration of more than 1 SDS during follow-up. Grote et al. [21] suggest referral of children with growth disorders at a growth deflection delta height of −1 SDS.

In 2015, Lambrechts et al. [27] considered a difference in SDS ≥0.5 for height-for-age to be clinically relevant in children treated with medium-chain triglyceride (MCT) KD. They found that the height-for-age declined in 30% of the children.

Recently, a retrospective study (Ferraris et al.) [28] was published on the impact of the KD on linear growth in 34 patients. The authors found that 20% of the children showed growth retardation at 12 months. In our study, using −1 SDS as a cut-off point, growth deceleration was observed in 9% (n: 4) of the patients.

These findings confirm that children with refractory epilepsy receiving KD treatment require careful growth monitoring. Close nutritional follow-up according to a protocol is essential to monitor the general nutritional status of these patients. A cut-off point of −1 SDS may be used as a parameter to evaluate the linear growth status for the subsequent fine-tuning of the diet, considering calorie-to-protein ratio, micronutrients, and ketogenic ratio.

The exact mechanism of growth delay during KD treatment is still unknown [29], but factors such as caloric restriction (energy-protein), metabolic acidosis (β-OHB levels), and low IGF-1 levels have been described [15,26,30]. Since in our series growth delay was found in the absence of calorie restriction, an additional mechanism affecting growth may be involved. In our study, the mean levels of ketosis were stable and within the therapeutic range. Peterson et al. [24] and Spulber et al. [26] showed that marked ketosis was related to delayed growth, while no impact on the height-for-age z-scores was observed in the presence of moderate ketosis. This mechanism may be associated with the metabolic changes caused by the diet, as suggested by other authors [26].

The identification of growth-related as well as protective factors will be helpful in clinical practice and in the counseling of families planning to extend the duration of the dietary treatment.

A limitation of this study is that confounding variables that may have caused delayed growth, such as different antiepileptic drugs, type of epileptic syndrome, or severity of neurologic dysfunction, were not considered in the evaluation. Additionally, we were not able to address the association between height velocity and hormonal growth status (IGF-1) before starting and while on the diet. Nevertheless, the strengths of the study are the prospective follow-up of the cohort and the use of cut-off points to assess growth delay.

Further studies evaluating the correlation between growth and KD treatment are needed to determine the mechanisms that underlie growth delay in children on KD.

## 5. Conclusions

The nutritional follow-up of these patients was helpful to improve overweight and thinness but could not avoid growth deceleration in some of them.

KD treatment resulted in a decline in linear growth without alterations in nutritional status measured by BMI in children with refractory epilepsy. Studies that explain the reason for this growth deceleration are lacking.

Since this diet is used as a long-term therapy for the treatment of different epileptic syndromes, careful monitoring of growth is necessary in children receiving the KD.

## Figures and Tables

**Table 1:** Baseline characteristics of children with intractable epilepsy on the ketogenic diet (KD).

Characteristics	Patients on the KD N45
Age year: month range	6.6 (0.8 to 17.3)
Male/Females	23/22
Ambulation, n	25
Classical KD 4:1/3:1/2:1	41/2/2
Orally fed/NGT-GTT	38/7
Mean AEDs	3.5
Responders/Non-responders (%)	74/26
Z-score height	−0.31 SDS ± 1.3
Z-score weight	−0.41 SDS ± 1.7
Z-score BMI	−0.26 SDS ± 1.5

AED: antiepileptic drugs, NGT: nasogastric tube, GTT: gastrostomy tube, BMI: Body Mass Index.

**Table 2:** Z-score delta changes of anthropometric measurements on patients on the ketogenic diet at 12 and 24 months.

Variables	Delta SDS 12 Months-SDS Baseline Median (max/min)	Delta SDS 24-SDS 12 Months Median (max/min)
Height	−0.32 (0.52/−1.46)	−0.36 (1.67/−1.56)
Weight	−0.24 (2.25/−1.42)	−0.28 (1.04/−1.40)
